# Ectopic thymoma in the paratracheal region of the middle mediastinum: a rare case report and literature review

**DOI:** 10.1186/s13104-018-3359-9

**Published:** 2018-04-25

**Authors:** Toshiki Yajima, Akira Mogi, Kimihiro Shimizu, Takayuki Kosaka, Toshiteru Nagashima, Yoichi Ohtaki, Kai Obayashi, Seshiru Nakazawa, Misaki Iijima, Yuka Yoshida, Junko Hirato, Hiroyuki Kuwano

**Affiliations:** 1 Division of General Thoracic Surgery, Integrative Center of General Surgery, Gunma University Graduate School of Medicine, Maebashi, Japan; 2 Department of Pathology, Gunma University Hospital, Maebashi, Japan

**Keywords:** Ectopic thymoma, Middle mediastinal tumor, Ectopic thymus, Paratracheal nodule

## Abstract

**Background:**

Thymomas generally arise from the thymus in the anterior mediastinum. Ectopic thymomas arising in the middle mediastinum are rare. We present a case of a thymoma arising from the ectopic thymic tissue in the right paratracheal region.

**Case presentation:**

The patient was a 67-year-old male who underwent an enhanced-computed tomography examination as preoperative staging for colon cancer. A 20-mm nodule in the right paratracheal region was found incidentally. Fluorodeoxyglucose (FDG) accumulation was detected in this solitary nodule by FDG-positron emission tomography, mimicking an enlarged, possibly malignant lymph node. The tumor was removed by thoracoscopic surgery, and a postoperative pathological diagnosis of type AB thymoma was made. Foci of ectopic thymic tissues were found adjacent to the thymoma. The patient was disease-free and without recurrence 2 years postoperatively.

**Conclusions:**

Including the present case, 13 cases of ectopic paratracheal thymoma have been reported in the English literature, all of which were found on the right side of the paratracheal region. Although ectopic thymomas in the paratracheal region are rare, thymomas may be considered as a differential diagnosis for a paratracheal nodule.

## Background

In general, thymomas originate from the thymus in the anterior mediastinum. Occasionally, an ectopic thymoma is found outside the anterior mediastinum, including in the neck, middle or posterior mediastinum, lung, and pleura [1]. Ectopic thymomas are thought to arise from the distributed thymic tissue that has failed to migrate into the anterosuperior mediastinum. We report a rare case of a thymoma arising from the ectopic paratracheal thymus in the middle mediastinum. Our search of pertinent English literature found that there were only 12 other cases of ectopic thymoma of the paratracheal region in the middle mediastinum, all of which were found on the right side of the paratracheal region [2–13]. Ectopic thymoma of the middle mediastinum may be considered as a differential diagnosis for a paratracheal nodule showing enhanced lesion on computed tomography (CT) or accumulated fluorodeoxyglucose (FDG) on positron emission tomography (PET).

## Case presentation

**Fig. 1:**
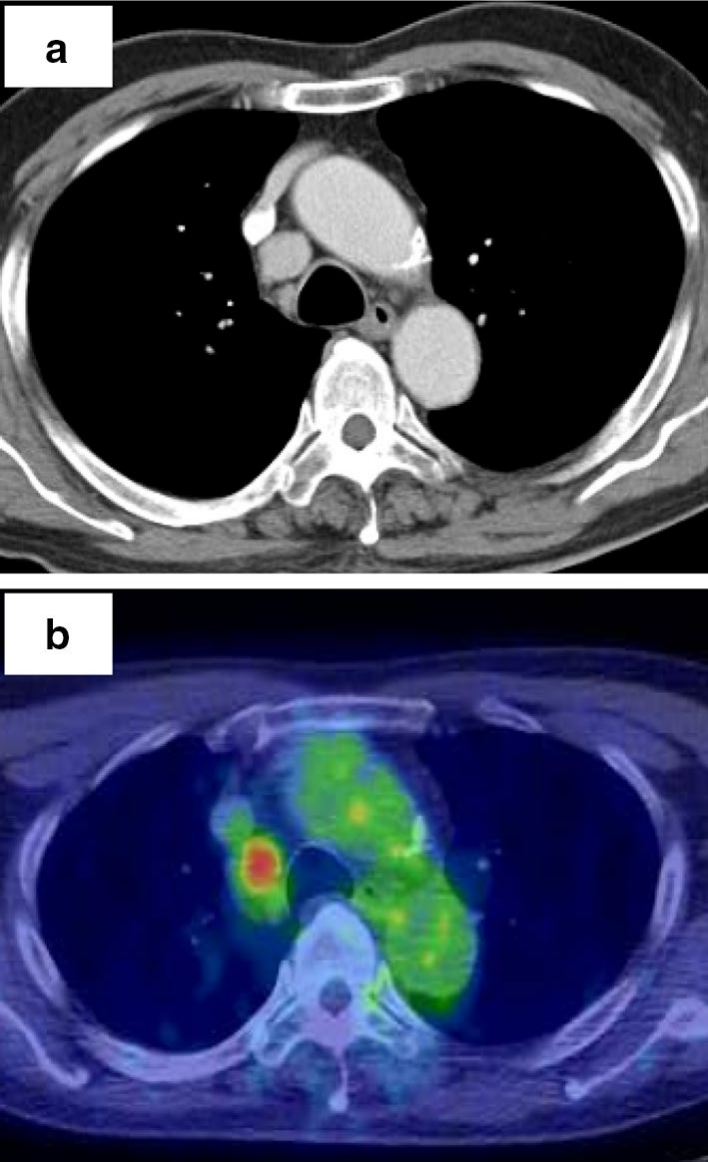
Ectopic thymoma detected in the right side of the paratracheal region by computed tomography (CT) and fluorodeoxyglucose-positron emission tomography (FDG-PET) examination. **a** Enhanced CT of the chest showed a solitary, well-defined nodule in the right paratracheal region. **b** The mediastinal tumor showed a marked increase in the accumulation of FDG

A 67-year-old male was admitted to our hospital for treatment of sigmoid colon cancer. Contrast-enhanced CT showed a homogenous enhanced nodule with a 20-mm diameter in the right paratracheal region. The nodule mimicked an enlarged paratracheal lymph node (LN) (Fig. 1a). During a FDG-PET examination for the preoperative staging of colon cancer, we detected FDG accumulation in the nodule, with a maximum standardized uptake value of 5.3 (Fig. 1b). The patient had no clinical symptoms. His and his family medical history was unremarkable. No abnormalities were found in blood tests, with the exception of the patient naturally having a high level of white blood cells. We determined that the nodule would be followed up after treatment for colon cancer. The patient underwent laparoscopic sigmoidectomy, and no abdominal LN metastasis (pSM, N0/stage I) was found. In a follow-up CT 3 months postoperatively, no difference was found in the diameter of the nodule in the middle mediastinum. We made a preliminary diagnosis of a malignant tumor, such as a malignant lymphoma or metastatic LN, because of the high FDG uptake, and the patient underwent surgical resection of the tumor without preoperative biopsy.

Tumor resection was performed by thoracoscopic surgery using four ports in the left lateral position. The tumor was located between the superior vena cava and trachea in the cranial side of the arch of azygos vein. After dissecting the mediastinal pleura, the tumor together with surrounding paratracheal LN was resected using a vessel sealing system, in accordance with LN dissection in lung cancer.

**Fig. 2:**
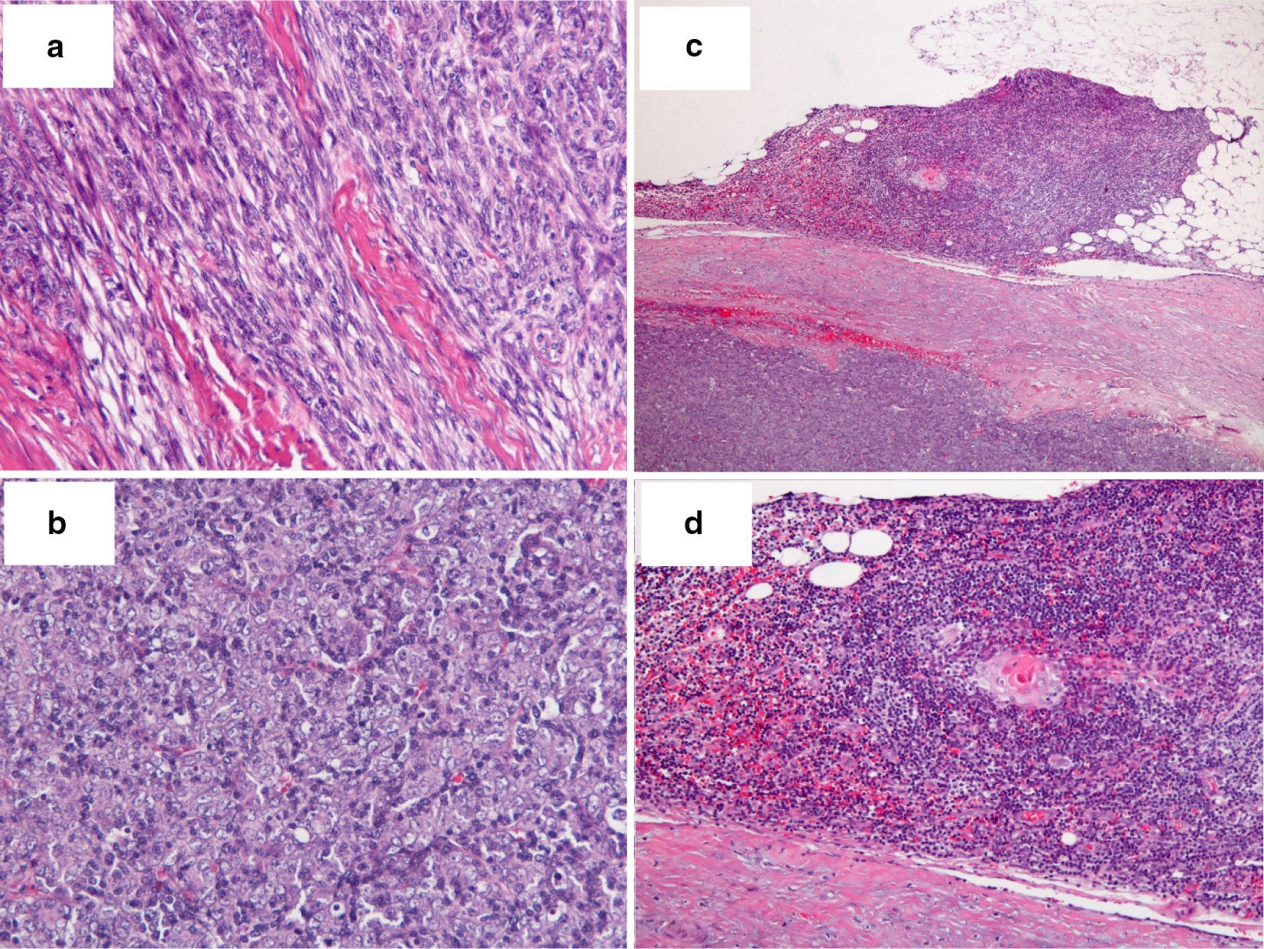
The histopathologic finding of the tumor revealed a type AB thymoma (World Health Organization classification) with the ectopic thymic tissue (hematoxylin and eosin staining). **a** In lymphocyte-poor areas, the oval- to spindle-shaped neoplastic cells were arranged in a fascicular pattern. **b** In lymphocyte-rich areas, polygonal-shaped neoplastic cells were arranged with vesicular nuclei in dense chromatin. In lymphocyte-rich areas, a diffuse proliferation of polygonal-shaped neoplastic cells was observed with vesicular nuclei and small nucleoli. **c**, **d** Foci of the ectopic thymic tissue with Hassall’s corpuscles were found adjacent to the tumor capsule. Original magnification: × 100 (**a**, **b**, **d**), × 40 (**c**)

The resected specimen measured 2.3 × 1.7 cm, and showed a well-encapsulated tumor with a lobulated appearance separated by fibrous bands. Microscopic examination showed that the specimen comprised lymphocyte-poor and lymphocyte-rich areas. In lymphocyte-poor areas, the oval- to spindle-shaped neoplastic cells were arranged in a fascicular pattern (Fig. 2a). A rosette-like structure was also present in these areas. In lymphocyte-rich areas, a diffuse proliferation of polygonal-shaped neoplastic cells with vesicular nuclei and small nucleoli was observed (Fig. 2b). A few mitotic figures were also noted. The tumor cells had invaded the capsule, but not the outside adipose tissue adjacent to the capsule. The ectopic thymic tissue foci were found adjacent to the thymoma capsule (Fig. 2c and d). Immunohistochemical staining showed that the thymic epithelial cells were positive for cytokeratin AE1/AE3 and negative for neuroendocrine markers including synaptophysin, CD56, and chromogranin A. In lymphocyte-rich areas, the small lymphocytes were positive for the immature T lymphocyte markers TdT, CD99, and CD1a. These findings were consistent with the World Health Organization (WHO) classification of type AB thymoma. The pathologic staging indicated stage II, based on the Masaoka staging system.

The postoperative course was uncomplicated. The patient received no adjuvant radiotherapy and survived with no recurrence at 2 years postoperatively.

## Discussion and conclusions

Thymomas are relatively rare tumors that arise from the thymic tissue and are typically located in the anterior mediastinum. Ectopic thymomas account for only 4% of all thymomas and have been reported in the neck, middle or posterior mediastinum, and the lung and pleura [1]. Middle mediastinal thymomas arising from the pleural and hilar adipose tissues are rare, and an ectopic thymoma in the paratracheal region in the middle mediastinum is extremely rare. Only 12 previous case reports exist in the English literature [2–13], and the inclusion of the present case brings this number to 13 cases in total.

Embryologically, the thymic tissue arises bilaterally from the third and possibly fourth bronchial pouches and migrates into the anterosuperior mediastinum. Ectopic thymomas are thought to arise from the distributed ectopic thymic tissue that has failed to migrate into the anterosuperior mediastinum. The thymic tissue exists in the adipose tissue surrounding the thymus and often has continuity with the pleural or hilar adipose tissue. Thymomas in the surrounding pleural and hilar adipose tissue are occasionally reported as a middle mediastinal thymoma [1]. The ectopic thymic tissue is also present in the retro innominate vein area [11]. Therefore, it is possible for thymoma in the paratracheal region to be located in the middle mediastinum. In the present case, we confirmed the presence of the ectopic thymic tissue adjacent to the thymoma in the paratracheal region by pathological examination, which suggested that the tumor arose from the ectopic thymic tissue behind the innominate veins.

A summary of the cases (13 reports including our case) of ectopic thymoma in the paratracheal region is presented in Table 1. Of the 13 patients, five were men and eight were women, with a mean age of 59.2 years (range 42–71 years). Almost all cases had no symptoms, with the exception of two patients with myasthenia gravis (MG). Nine cases were identified by plain film X-ray during a medical check-up. The mean size of the tumors was 5.2 cm (range 2.0–7.5 cm). Based on WHO classification, the histologic types of the tumors were type A (n = 2), type AB (n = 9), type B1 (n = 1), and type B3 (n = 1). The tumor stage was either stage I or II in all early cases, according to the Masaoka staging system. It is notable that the tumor location was in the right side of the paratracheal area in all cases. The reasons for tumors presenting in the right side of the trachea are not known and may include the ectopic thymic tissue being present in the right side of retro innominate vein area, embryonically or anatomically. A right-side paratracheal nodule may be considered as a possible thymoma, but the incidence of this is rare.

**Table 1:** A summary of the 13 cases of ectopic thymoma in the paratracheal region

Age (mean, range)	59.2 (42–71)
Gender, M/F	5/8
Symptoms, ±	2/11
Myasthenia gravis, ±	2/11
X-p/CT/PET	10/1/1
Tumor size (cm, mean, range)	5.2 (2.0–7.5)
Paratracheal site rt/lt	13/0
Preoperative diag. Benign/malignant	9/4
WHO classification A/AB/B1/B2/B3/C	2/9/1/0/1/0
Masaoka stage^a^ I/II/III/IVa/IVb	6/5/0/0/0

^a^Two patients: information not available

Differential diagnoses for paratracheal nodules include LN hyperplasia, lymphoma, metastatic LN, sarcoidosis, Castleman disease, and infectious disease. Neurogenic tumors and mediastinal goiters may also occur in the middle mediastinum. Endobronchial ultrasound-guided transbronchial needle aspiration (EBUS-TBNA) may be useful for diagnosing a paratracheal tumor. Although the main application of EBUS-TBNA is mediastinal LN staging for lung cancer, it has been found useful in the diagnosis of other diseases, such as sarcoidosis and lymphoma. Successful diagnosis and sub-classification using EBUS-TBNA has been reported in four cases of thymoma with an anterior mediastinal mass invading the paratracheal region [14]. Another report showed successful diagnosis of ectopic thymomas located in the paratracheal region in the middle mediastinum [10, 13]. However, diagnosis of thymomas by EBUS-TBNA is considered challenging as the tiny specimens obtained via EBUS-TBNA are not always sufficient for accurate diagnosis and sub-classification. In addition, there are reports of needle track seeding after biopsy of a thymoma [15]. In the present case, we found occasional FDG hot spots in preoperative FDG-PET imaging for a patient with colon cancer. Malignancy was possible because of high FDG uptake, and we recommended surgical tumor resection. Based on CT examination alone, we might have selected continuous follow-up examinations for an enlarged LN; additional PET examination was useful for the diagnosis of ectopic thymoma in the paratracheal region. Although ectopic thymoma in the middle mediastinum is extremely rare, occult thymoma may be diagnosed as enlarged LNs in the paratracheal region.

Surgical procedures for the treatment of ectopic thymomas are not well established. Thymothymomectomy is the standard surgical procedure for usual anterior mediastinum thymomas. However, almost all cases of paratracheal thymoma underwent simple tumor resection, based on a preoperative diagnosis of a benign tumor by imaging examination. A recent report suggested that simple resection of the tumor, including an adequate surgical margin from the tumor, was acceptable for the treatment of stage I thymoma, with regard to postoperative complications and prognosis [16]. Post-thymomectomy myasthenia gravis occurs occasionally after surgery in patients with usual thymomas and it has been reported that thymothymomectomy does not prevent postoperative MG [17]. Therefore, patients should be carefully monitored for MG after surgery. Simple resection of ectopic thymomas may be sufficient as treatment for patients without MG. In the cases reviewed, compression of the superior vena cava by paratracheal thymomas was usually found because of their anatomical location. However, no significant invasion was found in the adjacent mediastinal structure in any of the 13 cases. Almost all cases were low-risk thymomas according to WHO classification and were early stage thymoma in the Masaoka staging system. Video-assisted thoracoscopic surgery may be considered for resection of paratracheal thymomas. As it is difficult to obtain a sufficient surgical margin for a paratracheal thymoma, en bloc resection of the tumor may be necessary for complete resection, as in upper mediastinal LN dissection in lung cancer.

Thymomas are not often considered in the differential diagnosis of paratracheal nodules. However, because of the malignant potential and long-term survival of patients after complete resection, thymomas should be considered as a differential diagnosis for paratracheal tumors. Complete resection should be attempted at the time of the operation.

## Abbreviations

CT: computed tomography
FDG: fluorodeoxyglucose
PET: positron emission tomography
LN: lymph node
WHO: World Health Organization
MG: myasthenia gravis
EBUS-TBNA: endobronchial ultrasound-guided transbronchial needle aspiration
